# Electrical Storm/Refractory Ventricular Tachycardia

**DOI:** 10.21980/J8TS80

**Published:** 2024-04-30

**Authors:** Ashley R Tarchione, Amrita Vempati

**Affiliations:** * Kaiser Permanente San Diego Medical Center, Department of Emergency Medicine, San Diego, CA; ^ Creighton University School of Medicine Phoenix Program, Valleyhealth Medical Center, Department of Emergency Medicine, Phoenix, AZ

## Abstract

**Audience:**

This simulation case was created for emergency medicine (EM) residents at all levels of training.

**Background:**

Cardiac electrical storm (ES) is commonly defined as three or more episodes of sustained ventricular tachycardia, ventricular fibrillation, or three shocks from an implantable defibrillator within a 24 hour period.^1^ This can occur in up to 30–40% of patients with implantable defibrillators; however, it may also present in a wide variety of patients, including those with structural heart disease, myocardial infarction, electrolyte disturbances, and channelopathies.^2^,^3^ With each subsequent episode of ventricular arrhythmia, the arrhythmogenic potential of the heart may increase secondary to increased intracellular calcium dysregulation, myocardial injury, and increased endogenous release of catecholamines. The increased pain and catecholamine release from cardioversion/defibrillation and exogenous epinephrine during cardiac arrest further exacerbates ES.^2^ This carries a significant mortality risk of up to 12% in the first 48 hours.^3^

This case involves a basic knowledge of the Advanced Cardiac Life Support (ACLS) for ventricular tachycardia, both with and without a pulse, and the application of Sgarbossa criteria in a patient with an ST elevation myocardial infarction (STEMI) which makes it ideal for the PGY-1. However, the case quickly becomes refractory to the basic management prescribed in ACLS, requiring trouble shooting and quick thinking about deeper pathophysiology, a skill that is crucial for all emergency medicine physicians. There are multiple ways to troubleshoot this case, making for a good variety of discussion and recent literature review on the complexities of a relatively common arrhythmia, ventricular tachycardia.

**Educational Objectives:**

By the end of this simulation, learners should be able to: 1) recognize unstable ventricular tachycardia and initiate ACLS protocol, 2) practice dynamic decision making by switching between various ACLS algorithms, 3) create a thoughtful approach for further management of refractory ventricular tachycardia, 4) interpret electrocardiogram (ECG) with ST-segment elevation (STE) and left bundle branch block (LBBB), 5) appropriately disposition the patient and provide care after return of spontaneous circulation (ROSC), 6) navigate a difficult conversation with the patient’s husband when she reveals that the patient’s wishes were to not be resuscitated.

**Educational Methods:**

This simulation was performed using high-fidelity simulation followed by an immediate debriefing with nine learners who directly participated in the SIM and twenty-three residents, who were online observers via Zoom. This case was done during our conference day, and there were a total of approximately forty total learners comprised of medical students, PGY-1, PGY-2 and PGY-3 residents. There were several medical students who also observed via Zoom but were not surveyed, and the survey was sent to 32 learners. The case was run three separate times with each session consisting of three-four learners at the same level of training, with other learners in the same level of training observing via Zoom™ video platform. Since we can only have a team of three-four learners participate per group during simulation, the rest of the learners were observing the case and the debrief. There was one simulation instructor and one technician.

**Research Methods:**

We sent an online survey to all the participants and the observers after the debrief via surveymonkey.com. The survey collected responses to the following statements: (1) the case was believable, (2) the case had right amount of complexity, (3) the case helped in improving medical knowledge and patient care, (4) the simulation environment gave me a real-life experience and, (5) the debriefing session after simulation helped improve my knowledge. Likert scale was used to collect the responses.

**Results:**

A total of thirteen participants responded to the survey. One hundred percent of them either strongly agreed or agreed that the case was believable and that it helped in improving medical knowledge and patient care. Fifty-four percent strongly agreed, 38 percent agreed, and eight percent were neutral about the case having the right amount of complexity. Thirty one percent strongly agreed, 61 percent agreed, and eight percent were neutral about the case giving them real-life experience. All of them agreed that the debriefing session helped them improve their knowledge.

**Discussion:**

The high-fidelity simulation case was helpful with educating learners with ventricular tachycardia and fibrillation. Learners learned how to switch between various ACLS algorithms and how to manage a patient with refractory ventricular fibrillation. Learners enforced their knowledge in how to communicate with patient’s family members when the patient does not want resuscitation.

**Topics:**

Stable ventricular tachycardia, unstable ventricular tachycardia, refractory ventricular tachycardia, electrical storm, STEMI equivalents, medical simulation.

## USER GUIDE

[Table t1-jetem-9-2-s27]

**Table t1-jetem-9-2-s27:** 

List of Resources:	
Abstract	27
User Guide	29
Instructor Materials	31
Operator Materials	40
Debriefing and Evaluation Pearls	43
Simulation Assessment	47

**Learner Audience:**

Interns, Junior Residents, Senior Residents

**Time Required for Implementation:**

**Instructor Preparation:** 15 minutes

**Time for case:** 20 minutes

**Time for debriefing:** 10–15 minutes

**Recommended Number of Learners per Instructor:**

3

**Topics:**

Stable ventricular tachycardia, unstable ventricular tachycardia, refractory ventricular tachycardia, electrical storm, STEMI equivalents, medical simulation.

**Objectives:**

By the end of this simulation session, the learner will be able to:

1. Recognize unstable ventricular tachycardia and initiate ACLS protocol.
2. Demonstrate dynamic decision making by switching between various ACLS algorithms .
3. Discuss the management of refractory ventricular tachycardia.
4. Interpret electrocardiogram (ECG) with ST-segment elevation (STE) and right bundle branch block (LBBB).
5. Demonstrate the ability to navigate a difficult conversation with the patient’s husband when he reveals that the patient’s wishes were to not be resuscitated.

### Linked objectives and methods

Ventricular tachycardia is a common arrhythmia, but this case requires learners to think beyond typical management. The patient is initially in ventricular tachycardia; however, when she becomes confused early in the case, learners are expected to recognize her confusion as a sign of end organ malperfusion and recognize the unstable ventricular tachycardia and initiate ACLS protocol (Objective #1). While they are managing this, she becomes pulseless; thus, learners should be able to switch ACLS algorithms to the management of a pulseless patient (Objective #2). After the third shock delivery, learners should begin discussion and make a plan for management of refractory ventricular tachycardia (Objective #3). After return of spontaneous circulation (ROSC), learners should be able to interpret an ECG with right bundle branch block (LBBB) which meets criteria under the Modified Sgarbossa Criteria, and activate the cath lab (Objective #4). Learners should then provide appropriate MI and post-ROSC care (Objective #5). Learners will then need to communicate this plan to the family and navigate a difficult discussion, as the learners discover that the patient did not want to be resuscitated (Objective #6).

### Results and tips for successful implementation

This was implemented on three groups comprised of three to four emergency medicine residents. Since we can only have a small team (three to four) participate per group during simulation, the rest of the learners were observing the case and the debrief. Each group was comprised of learners from the same level of training: a group of PGY-1’s, PGY2’s, and PGY3’s. Other members of the resident classes that were not directly participating in the simulation watched the case in real-time through a Zoom platform. There were a total of 40 learners who participated directly and who observed on the Zoom platform. Separating the learners by classes proved very useful, since each level of learner took away something different from the case. As the simulation was run early in the year, those earlier in their training practiced more of the basic code management skills and changing strategies when working through a decompensating patient. More advanced learners were able to have time to work through more nuanced strategies to management of refractory ventricular tachycardia and have a more in-depth discussion about deviation from ACLS protocols and recent literature. Working within the 20-minute time frame, the amount and complexities of resuscitative efforts before ROSC were greater with more advanced learners. These flexibilities in management were built into the case. All learners participating either in-person or online were given pre- and post-quizzes to assess the success of the simulation.

After the simulation and debriefing session was complete, an online survey was sent via surveymonkey.com to all 32 participants. There were several medical students who also observed via Zoom but were not surveyed. The responses were collected on a Likert scale of 1 to 5 with 1 being “Strongly disagree” and 5 being “Strongly agree.” The survey collected responses to the following questions:

1. The case was believable.
2. The case had right amount of complexity.
3. The case helped in improving medical knowledge and patient care.
4. The simulation environment gave me a real-life experience.
5. The debriefing session after simulation helping improve my knowledge.

A total of 13 participants responded to the survey. The limited number of responses may be due to lack of interest in completing a survey. The results are shown as a graph below (Chart 1). Three out of 13 strongly agreed (23%) and 10/13 (77%) agreed that the case was believable. Seven out of 13 (54%) strongly agreed, 5/13 (38%) agreed, and 1/13 (8%) was neutral about the case having the right amount of complexity. Nine out of 13 (69%) strongly agreed and 4/13 (31%) agreed that the case helped in improving medical knowledge and care. Four out of 13 (31%) strongly agreed, 8/13 (61%) agreed, and 1/13 (8%) was neutral about the case giving them real-life experience. Finally, 11/13 (85%) strongly agreed and 2/13 (15%) agreed that the debriefing session helped in improving medical knowledge.[Fig f1-jetem-9-2-s27]

**Figure f1-jetem-9-2-s27:**
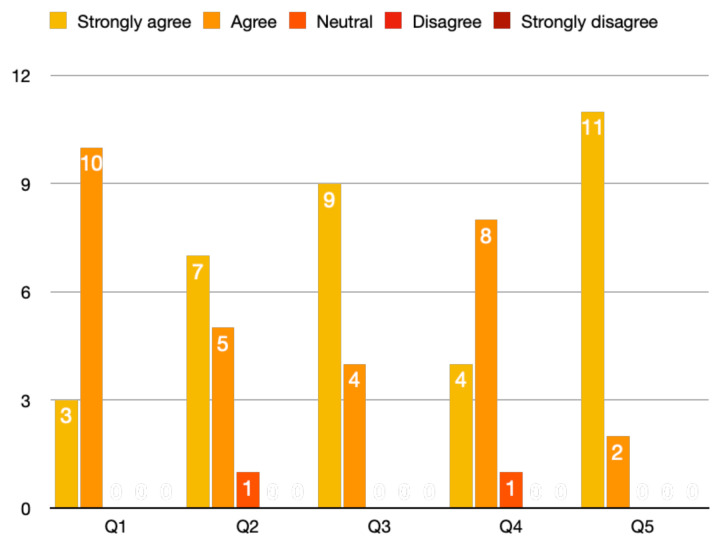


### Comments

*“Literally amazing sim case very helpful.”*

*“Great case, glad we went through it because I had never heard of it before.”*

*“I personally wasn’t at the conferences where we discussed electrical storm, so this was my first real experience learning about this in depth. After the case, I felt like I learned the topic fairly well and feel more comfortable approaching this case in real life.”*

*“Well run case that highlighted important topics with a good debrief afterward. A follow up reference to learn more about electrical storm, especially since we had a hard time finding a reference for the correct esmolol dosing during the case, would have been a helpful addition for follow up learning!”*

## Supplementary Information



## Figures and Tables

**Table t2-jetem-9-2-s27:** 

#1	Ventricular tachycardia ECG
#2	Chest X-ray with cardiomegaly (CXR)
#3	Post-ROSC ECG showing LBBB that meets the Modified Sgarbossa Criteria
#4	Complete blood count (CBC)
#5	Basic Metabolic Panel (BMP)
#6	Troponin
#7	Lactic acid

**Table t3-jetem-9-2-s27:** 

**Vitals:**	Heart Rate (HR) 180	Blood Pressure (BP) 150/90
	Respiratory Rate (RR) 20	Temperature (T) 37.0°C
	Oxygen Saturation (O_2_Sat) 97% on room air	

**Figure f2-jetem-9-2-s27:**
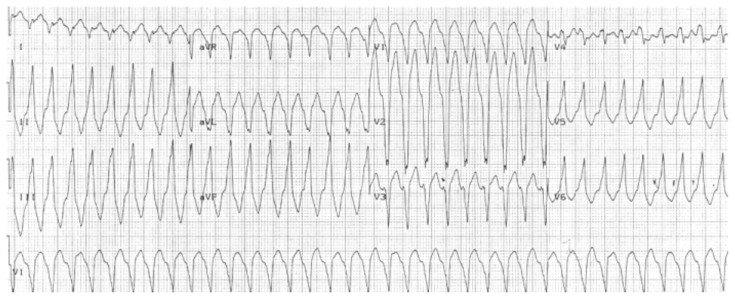


**Figure f3-jetem-9-2-s27:**
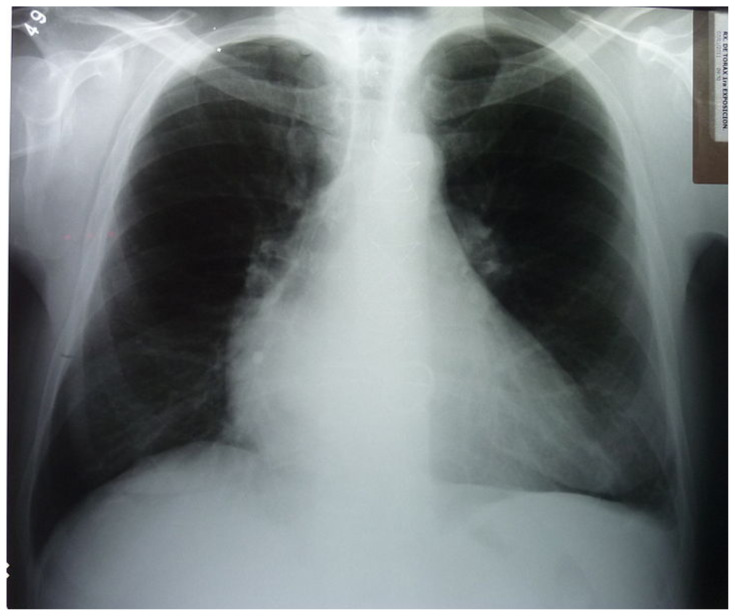


**Figure f4-jetem-9-2-s27:**
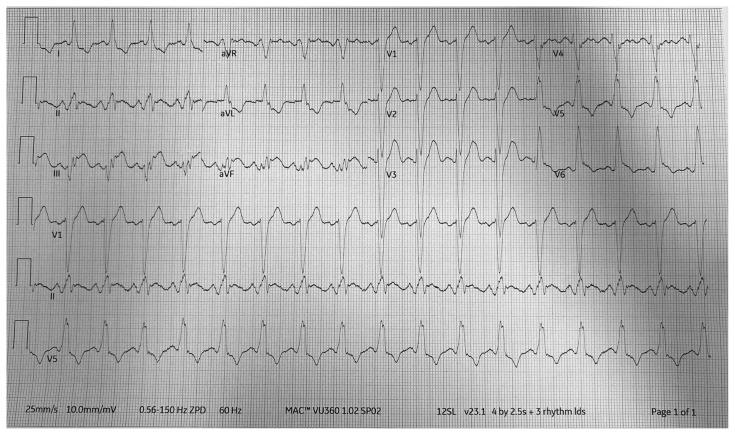


**Table t4-jetem-9-2-s27:** 

*Complete blood count (CBC)*	
White blood count (WBC)	13.3 × 1000/mm^3^
Hemoglobin (Hgb)	12.7 g/dL
Hematocrit (HCT)	35.7%
Platelet (Plt)	348 × 1000/mm^3^

**Table t5-jetem-9-2-s27:** 

*Basic metabolic panel (BMP)*	
Sodium	140 mEq/L
Potassium	3.9 mEq/L
Chloride	102 mEq/L
Bicarbonate (HCO_3_)	24 mEq/L
Blood Urea Nitrogen (BUN)	51 mg/dL
Creatinine (Cr)	0.71 mg/dL
Glucose	177 mg/dL
Calcium	8.7 mg/dL

**Table t6-jetem-9-2-s27:** 

*Troponin*	32 ng/L

**Table t7-jetem-9-2-s27:** 

*Lactic acid*	3.9 mmol/L

**Table t8-jetem-9-2-s27:** 

Magnesium	1.3 mEq/L

**Table t9-jetem-9-2-s27:** SIMULATION EVENTS TABLE:

Minute (state)	Participant action/ trigger	Patient status (simulator response) & operator prompts	Monitor display (vital signs)
0:00 Baseline	Team assesses vital signs, obtains history and assesses airway, breathing, and circulation Obtains ECG, places pads and initiates intravenous fluids Recognizes stable ventricular tachycardia and orders amiodarone	Patient is able to provide minimal history and has a pulse Before antiarrhythmics can be given, patient begins to decompensate by becoming more confused Before amiodarone was given, patient gets more altered	Rhythm: VT HR: 180/min BP: 150/80 RR: 16/min O 2 SAT: 95% T: 37.2° C
3:00 – 5:00	Team recognizes the worsening mental status and patient in unstable VT Performs synchronized cardioversion after adequately sedating the patient	Patient is harder to arouse and is confused but has pulses If participants do not notice worsening mental status, nurse may ask, “She is confused right now. Is there anything we can do to help her?”	Rhythm: VT HR: 184/min BP: 130/65 RR: 16/min O 2 SAT: 95% T: 37.2° C
5:00 – 8:00	Establishes endotracheal intubation Performs second synchronized cardioversion at higher energy	Patient is not responding anymore but has pulses If participants do not recognize the need for intubation, operator may decrease SpO2 reading. Nurse may note agonal respirations No change in the rhythm Triggers: -Second cardioversion→ go into Refractory Vtach state	Rhythm: VT HR: 187/min BP: 100/60 RR: 16/min O 2 SAT: 91% T: 37.2° C
8:00–11:00 Refractory ventricular tachycardia state	Learners will need to consider synchronized cardioversion for the third time	No change in the rhythm, and patient now intubated and has pulses Learners will need to be specific about the dosing of medications Consultants will not be available Modifiers: -If adenosine given: brief pause but no change on monitor	Rhythm: VT HR: 190/min BP: 90/50 RR: ventilator O2 SAT: 98%
11:00–15:00	Learners recognize pulseless VT and initiate ACLS protocol for pulseless VT Administer amiodarone Team may discuss esmolol and doing double sequential defibrillation	Patient is pulseless and intubated If participants do not notice change in status, nurse may say, “she has no pulses now.” Trigger: - Once amiodarone 300 mg bolus, go into ROSC	Rhythm: VT HR: 180/min BP: 0/0 RR: ventilator O2 SAT: 85%
15:00–18:00 ROSC	Interpret ECG Activate cath lab Initiate post-intubation sedation Give aspirin and initiate heparin	Patient is intubated, has pulses, and is not responding Cardiology will be available and will state that they will take the patient to the cath lab	Rhythm: sinus tachycardia HR: 110/min BP: 130/60 RR: vented O2 SAT: 100%
18:00–20:00 Case Completion	Learners will need to discuss care and next steps with family	Nursing cues, “Husband is here and wants to know what is going on.”	Rhythm: sinus tachycardia HR: 100/min BP: 140/80 RR: 16 O2 SAT: 98%

**Table t10-jetem-9-2-s27:** 

**Critical Actions:** Assess airway, breathing and circulation Obtain a thorough history and perform a complete physical examination Recognize VT and assess for stability Initiate and follow ACLS protocol for unstable VT with pulses Administer appropriate medication sedation and analgesia with cardioversion Assess for worsening mental status and establish endotracheal intubation Perform defibrillation during pulseless ventricular tachycardia according to ACLS Administer antiarrhythmic medication administration (amiodarone or lidocaine) Obtain post-ROSC ECG and interpret it correctly Activate cath lab Provide care after return of spontaneous circulation (ROSC) Appropriately disposition the patient to the ICU Notify the patient’s family about the course and address their concerns	0:00

**Table t11-jetem-9-2-s27:** 

	Milestone	Did not achieve level 1	Level 1	Level 2	Level 3
1	**Emergency Stabilization (PC1)**	□ Did not achieve Level 1	□ Recognizes abnormal vital signs	□ Recognizes an unstable patient, requiring intervention Performs primary assessment Discerns data to formulate a diagnostic impression/plan	□ Manages and prioritizes critical actions in a critically ill patient Reassesses after implementing a stabilizing intervention
2	**Performance of focused history and physical (PC2)**	□ Did not achieve Level 1	□ Performs a reliable, comprehensive history and physical exam	□ Performs and communicates a focused history and physical exam based on chief complaint and urgent issues	□ Prioritizes essential components of history and physical exam given dynamic circumstances
3	**Diagnostic studies (PC3)**	□ Did not achieve Level 1	□ Determines the necessity of diagnostic studies	□ Orders appropriate diagnostic studies. Performs appropriate bedside diagnostic studies/procedures	□ Prioritizes essential testing Interprets results of diagnostic studies Reviews risks, benefits, contraindications, and alternatives to a diagnostic study or procedure
4	**Diagnosis (PC4)**	□ Did not achieve Level 1	□ Considers a list of potential diagnoses	□ Considers an appropriate list of potential diagnosis May or may not make correct diagnosis	□ Makes the appropriate diagnosis Considers other potential diagnoses, avoiding premature closure
5	**Pharmacotherapy (PC5)**	□ Did not achieve Level 1	□ Asks patient for drug allergies	□ Selects an medication for therapeutic intervention, consider potential adverse effects	□ Selects the most appropriate medication and understands mechanism of action, effect, and potential side effects Considers and recognizes drug-drug interactions
6	**Observation and reassessment (PC6)**	□ Did not achieve Level 1	□ Reevaluates patient at least one time during case	□ Reevaluates patient after most therapeutic interventions	□ Consistently evaluates the effectiveness of therapies at appropriate intervals
7	**Disposition (PC7)**	□ Did not achieve Level 1	□ Appropriately selects whether to admit or discharge the patient	□ Appropriately selects whether to admit or discharge Involves the expertise of some of the appropriate specialists	□ Educates the patient appropriately about their disposition Assigns patient to an appropriate level of care (ICU/Tele/Floor) Involves expertise of all appropriate specialists
9	**General Approach to Procedures (PC9)**	□ Did not achieve Level 1	□ Identifies pertinent anatomy and physiology for a procedure Uses appropriate Universal Precautions	□ Obtains informed consent Knows indications, contraindications, anatomic landmarks, equipment, anesthetic and procedural technique, and potential complications for common ED procedures	□ Determines a back-up strategy if initial attempts are unsuccessful Correctly interprets results of diagnostic procedure
20	**Professional Values (PROF1)**	□ Did not achieve Level 1	□ Demonstrates caring, honest behavior	□ Exhibits compassion, respect, sensitivity and responsiveness	□ Develops alternative care plans when patients’ personal beliefs and decisions preclude standard care
22	**Patient centered communication (ICS1)**	□ Did not achieve level 1	□ Establishes rapport and demonstrates empathy to patient (and family) Listens effectively	□ Elicits patient’s reason for seeking health care	□ Manages patient expectations in a manner that minimizes potential for stress, conflict, and misunderstanding. Effectively communicates with vulnerable populations, (at risk patients and families)
23	**Team management (ICS2)**	□ Did not achieve level 1	□ Recognizes other members of the patient care team during case (nurse, techs)	□ Communicates pertinent information to other healthcare colleagues	□ Communicates a clear, succinct, and appropriate handoff with specialists and other colleagues Communicates effectively with ancillary staff

## INSTRUCTOR MATERIALS

**Case Title:** Electrical Storm/Refractory Ventricular Tachycardia

**Case Description & Diagnosis (short synopsis):** A 64-year-old female is brought to the ED with fatigue and a syncopal event. On arrival, she will be found to be in monomorphic ventricular tachycardia with associated chest discomfort with pulse. Despite cardioversion, the patient will convert back into monomorphic VT without pulse. After defibrillation attempts, patient will remain in VT and will require antiarrhythmics. After antiarrhythmic administration, ROSC will be achieved. Electrocardiogram (ECG) interpretation will reveal STEMI with LBBB meeting Sgarbossa criteria. Eventually cardiology consultation for definitive reperfusion and possible automated, implantable, cardioverter defibrillator (AICD) in the cardiac catheterization (cath) lab. When family arrives, the team must discuss the patient’s care and next steps with the patient’s husband.

**Equipment or Props Needed:**

– High-fidelity simulator
– Airway supplies:
  ○ Nasal cannula
  ○ Non-rebreather mask
  ○ Laryngoscope and blades
  ○ Endotracheal tube and stylet
  ○ Bag-valve mask
– IV supplies:
  ○ Two 18g angiocatheters
  ○ IV tubing
  ○ Normal saline
  ○ Lactated ringers
– Medications:
  ○ Etomidate
  ○ Succinylcholine
  ○ Rocuronium
  ○ Epinephrine
  ○ Sodium bicarbonate
  ○ Magnesium sulfate
  ○ Potassium Chloride
  ○ Amiodarone
  ○ Lidocaine
  ○ Esmolol
– Cardiac monitor
– Defibrillator
– Gloves
– Bedside Ultrasound

**Actors needed:**

Nurse

Husband

**Stimulus Inventory:** [Table t2-jetem-9-2-s27]

**Background and brief information:** Patient is a 64-year-old female presenting for fatigue and chest pain.

**Initial presentation:** Patient appears fatigued and mildly uncomfortable but is awake and alert.

**How the scene unfolds:** This patient is in monomorphic VT with associated ischemic chest pain. Learners should assess for the stability of the patient and determine that the patient needs cardioversion. After the first shock, the patient will have a brief conversion to normal sinus rhythm, but will then quickly convert back into monomorphic VT. Learners will need to cardiovert again while making sure to treat the pain. After that, the patient will be altered and will remain in VT, and learners will need to cardiovert for the third time. After the third cardioversion, patient will stop responding completely and will lose pulses. Learners will need to assess for airway and establish a secure airway in addition to switching to pulseless VT ACLS algorithm. Learners will need to defibrillate and administer an antiarrhythmic, namely amiodarone. After amiodarone administration, patient will achieve ROSC. Learners may discuss doing double sequential defibrillation and esmolol. After ROSC, learners will need to obtain ECG which shows a LBBB with STEMI, and they will need to recognize the STEMI and activate the cath lab. Patient’s husband will reveal that she never wanted to be resuscitated. Learners will need to discuss with the patient’s family and navigate a difficult conversation.

**Critical actions:**

1. Assess airway, breathing and circulation
2. Obtain a thorough history and perform a complete physical examination
3. Recognize VT and assess for stability
4. Initiate and follow ACLS protocol for unstable VT with pulses
5. Administer appropriate medication sedation and analgesia with cardioversion
6. Assess for worsening mental status and establish endotracheal intubation
7. Perform defibrillation during pulseless ventricular tachycardia according to ACLS
8. Administer antiarrhythmic medication administration (amiodarone or lidocaine)
9. Obtain post-ROSC ECG and interpret it correctly
10. Activate cath lab
11. Provide care after return of spontaneous circulation (ROSC)
12. Appropriately disposition the patient to the ICU
13. Notify the patient’s family about the course and address their concerns

**Case Title:** Electrical Storm/Refractory Ventricular Tachycardia

**Chief Complaint:** “Passed out”[Table t3-jetem-9-2-s27]

**General Appearance:** Tired-appearing, alert elderly female

**Primary Survey:**

- **Airway:** Protecting airway, speaking in full, clear sentences
- **Breathing:** Equal chest rise, bilateral breath sounds
- **Circulation**: 2+ bilateral radial pulses

**History:**

- **History of present illness:** On and off throughout the day, the patient has been feeling lighted-headed and tired. She passed out while cooking shortly before arrival, prompting her daughter to call EMS. She feels a mild amount of chest discomfort now which she describes as a substernal pressure. She still feels generally very weak. She does not believe this feels like her previous MIs, since she does not remember being so tired with those events.
- **Past medical history:** Ischemic cardiomyopathy (if asked: believes last EF was ~40%, but it has been a few years since she has seen her cardiologist), myocardial infarction five years prior, hypertension (HTN), hyperlipidemia
- **Past surgical history:** Cardiac stent placement 5 years ago
- **Patients medications:** ASA 81 mg, atorvastatin, lisinopril, metoprolol, furosemide
- Allergies: None
- **Social history:** Patient has a history of smoking with a 30 pack year history. She also drinks occasionally.
- **Family history:** Father with MI and stroke, mother with HTN and dementia

**Secondary Survey/Physical Examination:**

- **General Appearance**: Tired and uncomfortable appearing and clutching her chest. Easily arousable and appropriate
- **HEENT**
  ○ Head: within normal limits
  ○ Eyes: within normal limits
  ○ Ears: within normal limits
  ○ Nose: within normal limits
  ○ Throat/oropharynx: within normal limits
- **Neck:** Within normal limits
- **Heart:** Tachycardic, no murmurs, rubs or gallops
- **Lungs:** Within normal limits
- **Abdominal/GI:** Within normal limits
- **Genitourinary:** Within normal limits
- **Rectal:** Within normal limits
- **Extremities:** 1+ bilateral LE pitting edema
- **Back:** Within normal limits
- **Neuro:** Within normal limits. Patient is alert and oriented x 2 and is oriented to person and place but not time.
- **Skin:** Within normal limits
- **Lymph:** Within normal limits
- **Psych:** Within normal limits

*Electrocardiogram # 1* – Ventricular tachycardia

Ksheka at English Wikipedia. RVOT Tachycardia. In: Wikimedia Commons.

https://commons.wikimedia.org/wiki/File:RVOT_Tachycardia.png. CC BY-SA 3.0[Fig f2-jetem-9-2-s27]

*CXR –* CXR with cardiomegaly

SCiardullo. Enfermedad Mitral In: Wikimedia Commons.

https://commons.wikimedia.org/wiki/File:Enfermedad_Mitral.JPG. CC BY-SA 3.0[Fig f3-jetem-9-2-s27]

*Electrocardiogram # 2:* Post-ROSC ECG showing LBBB that meets the Modified Sgarbossa

Criteria for a STEMI equivalent

Image source: Courtesy of Kelsey Keeling, DO[Fig f4-jetem-9-2-s27][Table t4-jetem-9-2-s27][Table t5-jetem-9-2-s27][Table t6-jetem-9-2-s27][Table t7-jetem-9-2-s27][Table t8-jetem-9-2-s27]

## OPERATOR MATERIALS

[Table t9-jetem-9-2-s27]

**Diagnosis:**

Refractory ventricular tachycardia, STEMI, cardiopulmonary arrest

**Disposition:**

ICU

## DEBRIEFING AND EVALUATION PEARLS

### Electrical Storm/Refractory Ventricular Tachycardia

#### Unstable Monomorphic Ventricular Tachycardia

Unstable wide ventricular tachycardia is defined by the development of hypotension, altered mental status, ischemic chest discomfort, and/or acute heart failure. This requires emergent synchronized cardioversion. If the rhythm is narrow, adenosine may be considered, but the patient in this case had a wide complex tachycardia; thus, it would not be appropriate. If the patient is stable with a wide complex tachyarrhythmia, such as our patient presented at the start of the case, they can be given one of the following antiarrhythmics: procainamide 20/50 mg/min until arrhythmia ceases, amiodarone 150 mg over 10 minutes with a repeat bolus as needed, or 100 mg of sotalol over 5 minutes. Because our patient presented with findings of likely congestive heart failure (CHF) exacerbation, procainamide would not have been an appropriate choice.^5^

#### Pulseless Monomorphic Ventricular Tachycardia

An adult patient with pulseless ventricular tachycardia requires immediate cardiac defibrillation at 120-200J, with epinephrine administration after the second defibrillation and an antiarrhythmic administration after the third episode of defibrillation.^6^ Amiodarone is often the first line antiarrhythmic, resulting in termination of approximately 40% of ventricular arrhythmias as a solo agent through its effects on potassium channel blockade, sodium channel inhibition, L-type calcium channels, and sympathetic blockade.^2^ Lidocaine, a class I antiarrhythmic, may also be utilized, although it has been shown to be less effective than amiodarone. However, there is a potential modest benefit in patients with ventricular tachycardia secondary to ischemia when administered prophylactically after ROSC was achieved in patients with pulseless ventricular arrhythmias.^1^,^5^

Correction of underlying electrolyte derangements remains crucial to management of all arrhythmias. Additionally, it is vital to ensure that this is not polymorphic ventricular tachycardia, as the management is different altogether.^1^

#### Electrical Storm

Electrical storm is commonly defined as three or more episodes of sustained ventricular tachycardia or ventricular fibrillation requiring defibrillation or cardioversion, respectively, or three shocks from an implantable defibrillator within a 24-hour period.^1^,^2^,^3^,^4^ With each cardioversion or defibrillation episode, the potential for ongoing arrhythmia increases secondary to myocyte calcium dysregulation, injury, and increased endogenous release of circulating catecholamines. Exogenous epinephrine during cardiac arrest further exacerbates electrical storm.^2^ This carries a significant mortality risk of up to 14% in the first 48 hours.^3^

Electrical storm happens most commonly in patients with structural heart disease, with an ejection fraction (EF) of <25% considered a strong predictor.^4^ Fibrosis and scarring may lead to conduction blocks which result in abnormal electrical pathways in the heart.^2^,^3^ Precipitants may be reversible such as myocardial infarction, heart failure exacerbation, drug toxicity, thyrotoxicosis, electrolyte derangement, or QT prolongation, although greater than 80% of cases have no identifiable trigger.^2^,^3^,^4^ Hyperactivity of the sympathetic nervous system remains an important trigger for ventricular arrhythmia, which becomes further exacerbated by the loss of parasympathetic vagal tone during cardiac arrest.^4^

#### Management of Electrical Storm

Appropriate history taking, as is crucial in most cases, is vital here to address possible underlying causes of ES. Electrolyte derangements should be corrected immediately. An ECG should be immediately obtained, not only to identify ventricular arrhythmias, but also to evaluate for the possibility of a supraventricular tachycardia with aberrant conduction. However, all unstable patients should always be presumed to be in ventricular tachycardia.^3^

Standard ACLS pathways as delineated above should initially be followed. However, after failed management with three attempts at cardioversion or defibrillation, the patient is likely experiencing electrical storm and may be refractory to typical management. Although there is no widely accepted protocol for the management of the refractory patient, most literature on ES focuses on the management of sympathetic overdrive.

The first priority to control catecholamine release and further potential the arrhythmogenic state is to ensure that the patient and sedation are adequately controlled. In the awake patient, early intubation may assist in blunting the pain response and assist with arrhythmia control. One case report has found Propofol as a solo agent to be a successful agent in blunt refractory tachycardia, with the caveat that patients may need additional vasoconstrictor support.^1^ In patients experiencing ES with an implantable, cardioverter defibrillator (ICD), the ICD should be turned off by placing a magnet over the device. The pain experiences from repeated shocks from an ICD or cardioversion further heightens sympathetic drive.^4^

Overstimulation of beta receptors from increased catecholamines is thought to be the primary mechanism by which ventricular tachycardia becomes refractory. After adequately controlling for the pain, which leads to this catecholamine surge, it is important to blunt the response on the beta receptors themselves. Amiodarone remains the best first line agent for all ventricular arrhythmias, as discussed above, and electrical storm is no exception. All patients, even those chronically on amiodarone, should be given a bolus.^5^ If amiodarone fails as a solo agent, betablockers should be attempted. The combination of amiodarone and beta blockade in electrical storm has demonstrated higher rates of survival.^6^ Non-selective beta blockers such as propranolol may be superior in blunting sympathetic drive, due to the downregulation of B1 receptors in heart failure.^7^ It is important to acknowledge that blunting the beta adrenergic response is counterintuitive to the routine administration of epinephrine. Current ACLS guidelines provide a weak recommendation and very low-certainty evidence for epinephrine administration during ventricular tachycardia.^5^ However, because this is still the current guideline, holding epinephrine in favor of beta blockade will need to be clearly communicated to your team.

Double sequential defibrillation has also been suggested as a means to terminate refractory ventricular rhythms. In double sequential defibrillation, two defibrillators are used to deliver simultaneous or rapid sequential shocks. It is theorized that the increased energy and possibly the vector change of the energy delivered could contribute to improved defibrillation outcomes. One pilot RCT demonstrated promising outcomes with ventricular fibrillation termination and ROSC; however, a larger randomized control trail is still pending to formally evaluation survival and other benefits.^8^,^9^

#### Modified Sgarbossa Criteria

Recognizing occlusive myocardial infarctions in patients with left and right bundle branch blocks can be challenging because the conventional findings of one mm elevation in two anatomically contiguous leads does not apply. The modified Sgarbossa criteria portends that an occlusive MI can be detected on an ECG with LBBB when there is a concordant ST elevation or depression of ≥1 mm in leads with a positive QRS complex, concordant ST depression of ≥1 mm in leads V1-V3, or a discordance ST elevation ≥ 25% of the proceeding QRS complex. When analyzing an ECG for the presence of ischemia, the application of the Modified Sgarbossa criteria will identify coronary occlusion with a sensitivity of 91% and a specificity of 90%.^10^

#### Breaking Bad News in the Emergency Department

At the end of this case, learners need to discuss the patient’s decompensation and critical status with the patient’s husband, who is understandably distraught. When delivering bad news in the emergency department, there are a variety of tools and techniques to help guide the ED physician, with one of the most popular being the SPIKES mnemonic:

1. Setting up: Have the encounter in a private, quiet place with a small group of significant others.
2. Perception: Ask what those present know about the current situation.
3. Invitation: Ask if all who are present would like the full details of the patient’s condition.
4. Knowledge: Deliver the knowledge of the patient’s condition in a straight-forward way, while avoiding unnecessary bluntness or harsh phases. Use simple language.
5. Emotions: Pause to observe emotions (anger, sadness, shock), than validate those emotions and help them to identify the reason for them (eg, “I see you are angry; that is normal when you hear about a loved one being sick.”). Express empathy.
6. Strategy and Summary: Identify next steps and care. This can be done with social work or the next team of physicians that will be assuming care when the patient leaves the emergency department.

Providers that have used this protocol report increased confidence in delivering bad news.^11^

## SIMULATION ASSESSMENT

*Electrical Storm/Refractory Ventricular Tachycardia*

Learner: _________________________________________

***Assessment Timeline***

This timeline is to help observers assess their learners. It allows observer to make notes on when learners performed various tasks, which can help guide debriefing discussion.[Table t10-jetem-9-2-s27]

**Critical Actions:**

□ Assess airway, breathing and circulation
□ Obtain a thorough history and perform a complete physical examination
□ Recognize VT and assess for stability
□ Initiate and follow ACLS protocol for unstable VT with pulses
□ Administer appropriate medication sedation and analgesia with cardioversion
□ Assess for worsening mental status and establish endotracheal intubation
□ Perform defibrillation during pulseless ventricular tachycardia according to ACLS
□ Administer antiarrhythmic medication administration (amiodarone or lidocaine)
□ Obtain post-ROSC ECG and interpret it correctly
□ Activate cath lab
□ Provide care after return of spontaneous circulation (ROSC)
□ Appropriately disposition the patient to the ICU
□ Notify the patient’s family about the course and address their concerns

**Summative and formative comments:**

**Milestones assessment:** [Table t11-jetem-9-2-s27]
