# Supplementary material for: Electrical Storm/Refractory Ventricular Tachycardia
Source: J Educ Teach Emerg Med. 2024 Apr 30;9(2):S27–54. doi: 10.21980/J8TS80 (PMC11068320; doi:10.21980/J8TS80)
Supplement: Supplementary file 1 [file jetem-9-2-S27-supp1.pptx]

## Slide 1
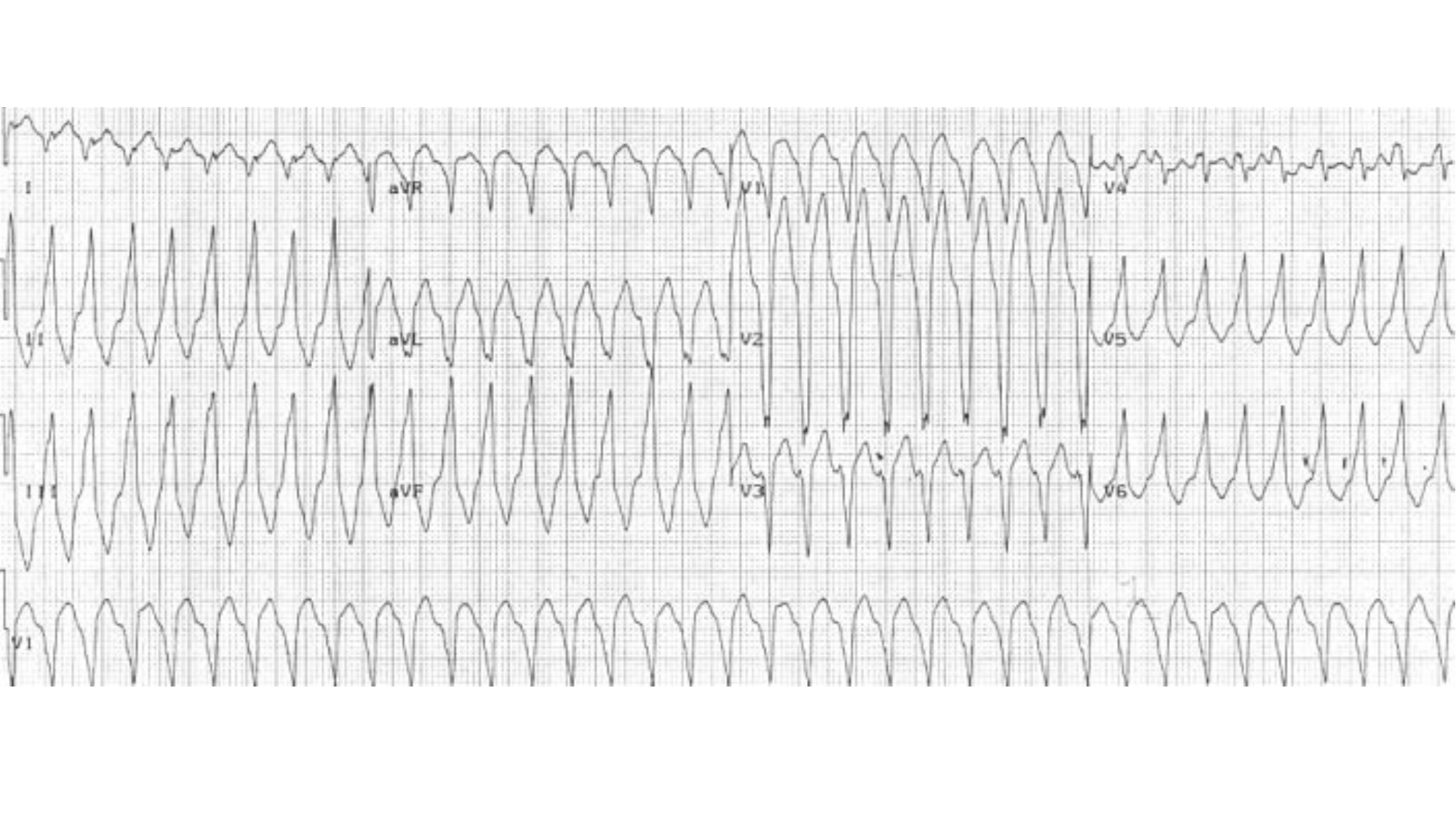

## Slide 2
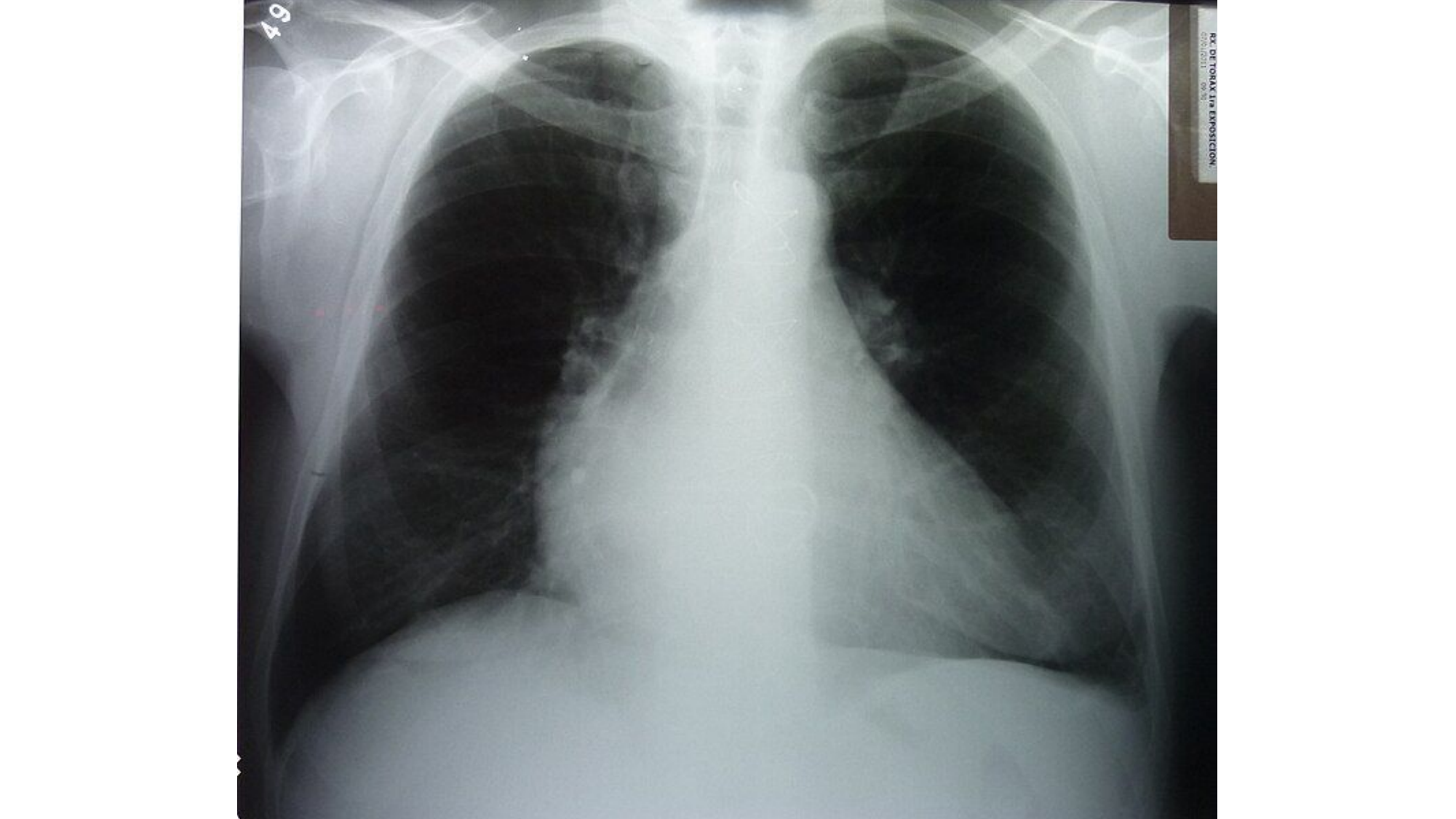

## Slide 3
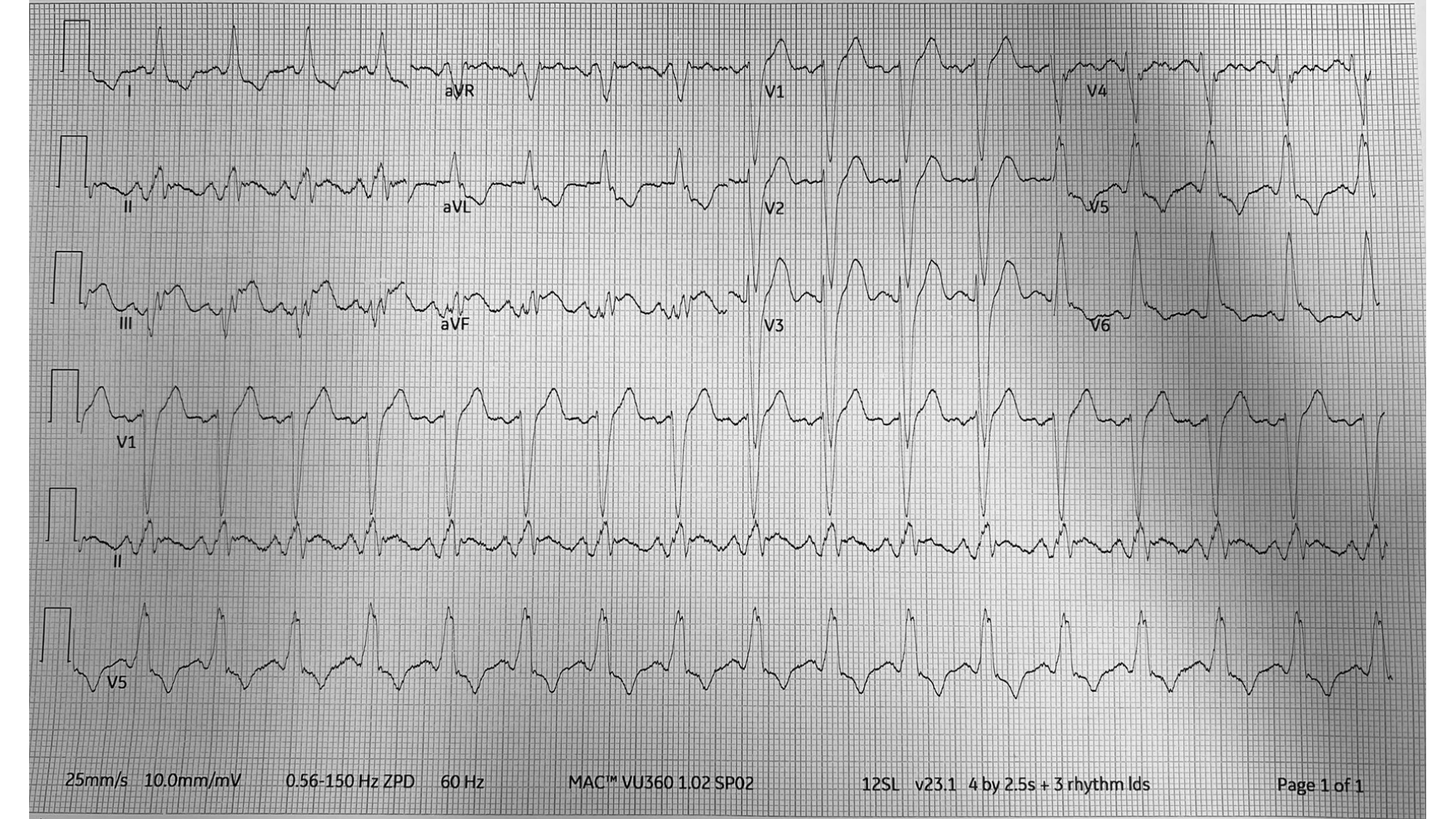

## Slide 4
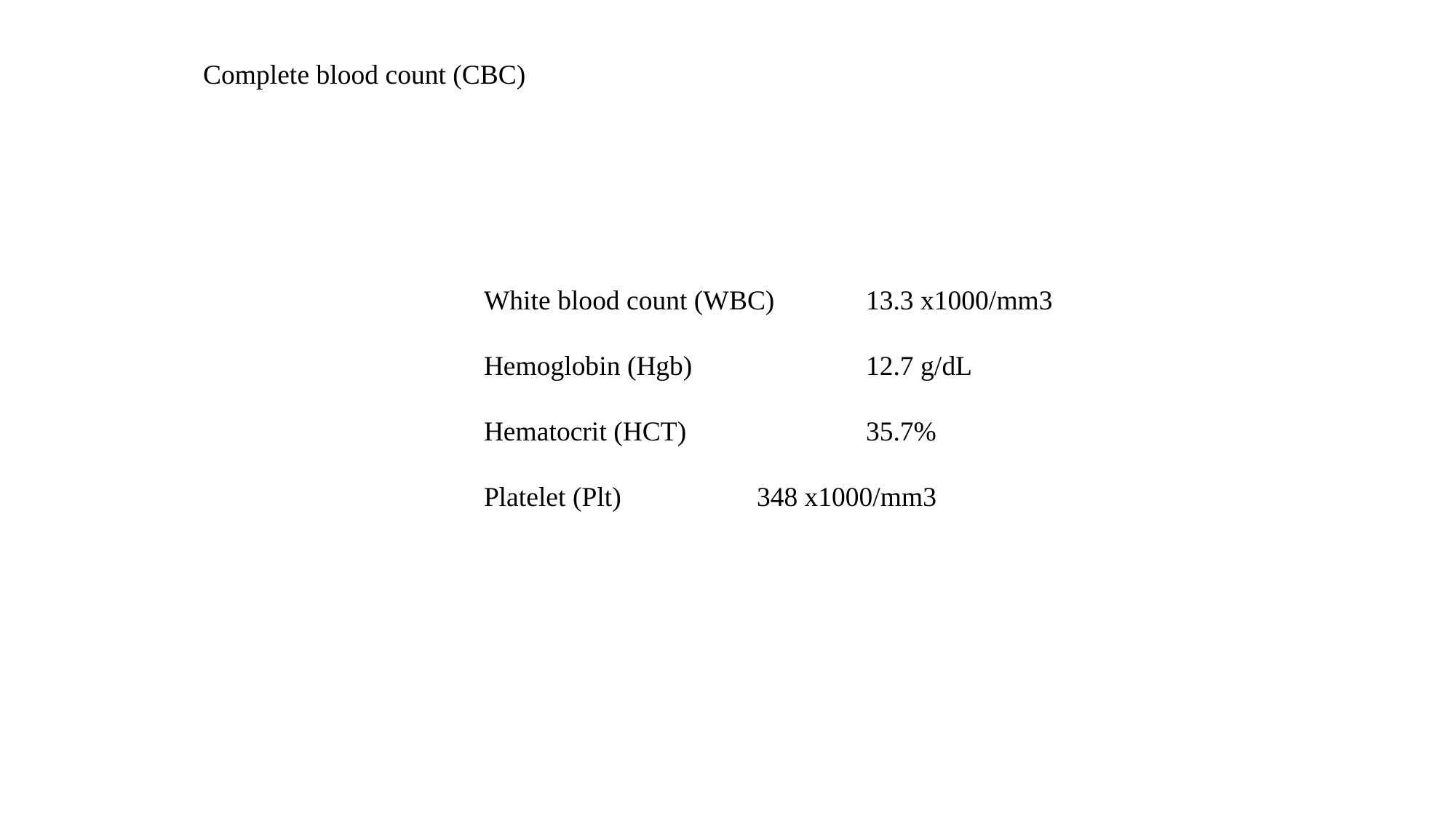

Complete blood count (CBC)
White blood count (WBC) 	13.3 x1000/mm3
Hemoglobin (Hgb) 		12.7 g/dL
Hematocrit (HCT) 		35.7%
Platelet (Plt) 		348 x1000/mm3

## Slide 5
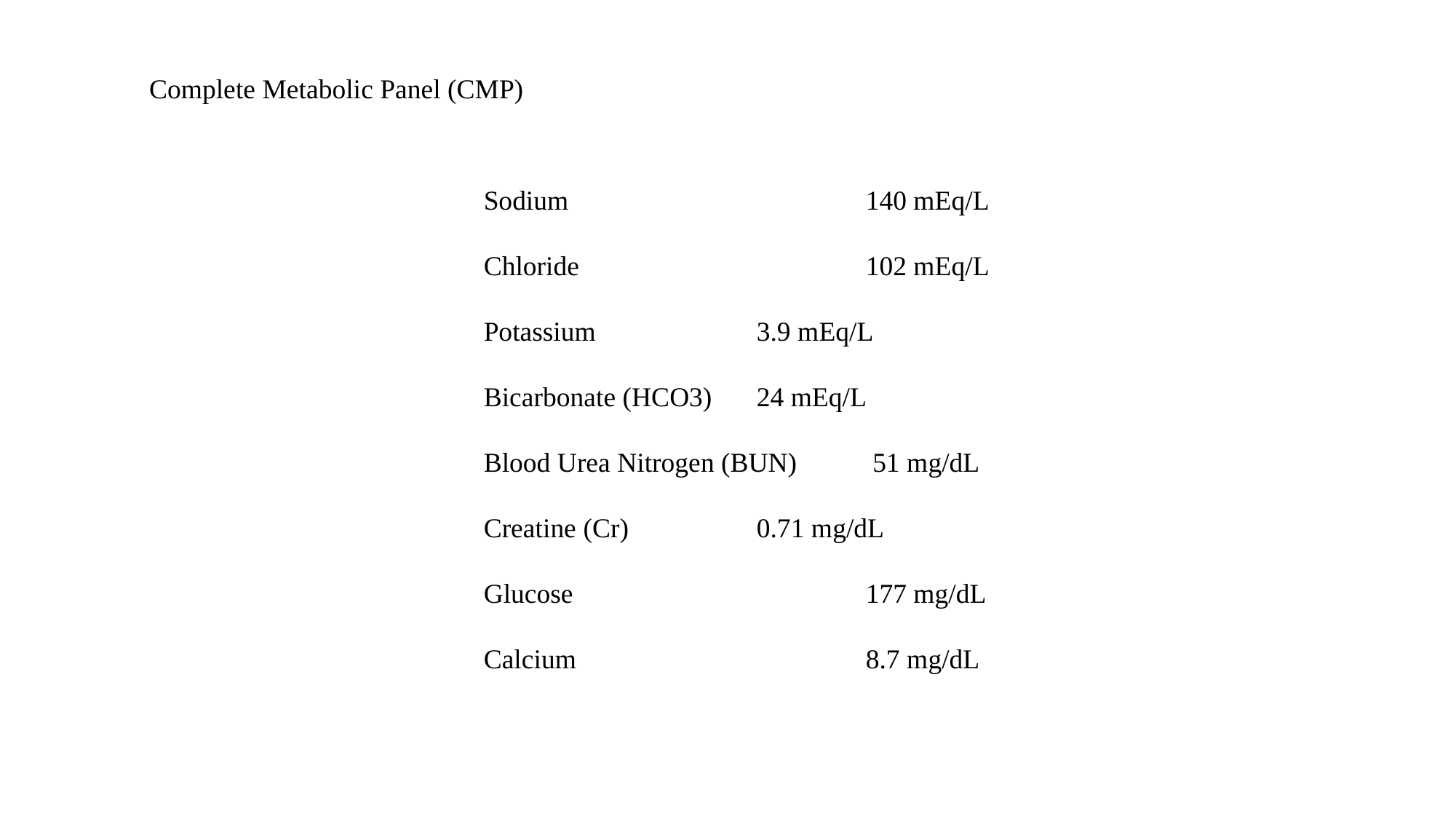

Complete Metabolic Panel (CMP)
Sodium 			140 mEq/L
Chloride 			102 mEq/L
Potassium 		3.9 mEq/L
Bicarbonate (HCO3)	24 mEq/L
Blood Urea Nitrogen (BUN)	 51 mg/dL
Creatine (Cr)		0.71 mg/dL
Glucose 			177 mg/dL
Calcium 			8.7 mg/dL

## Slide 6
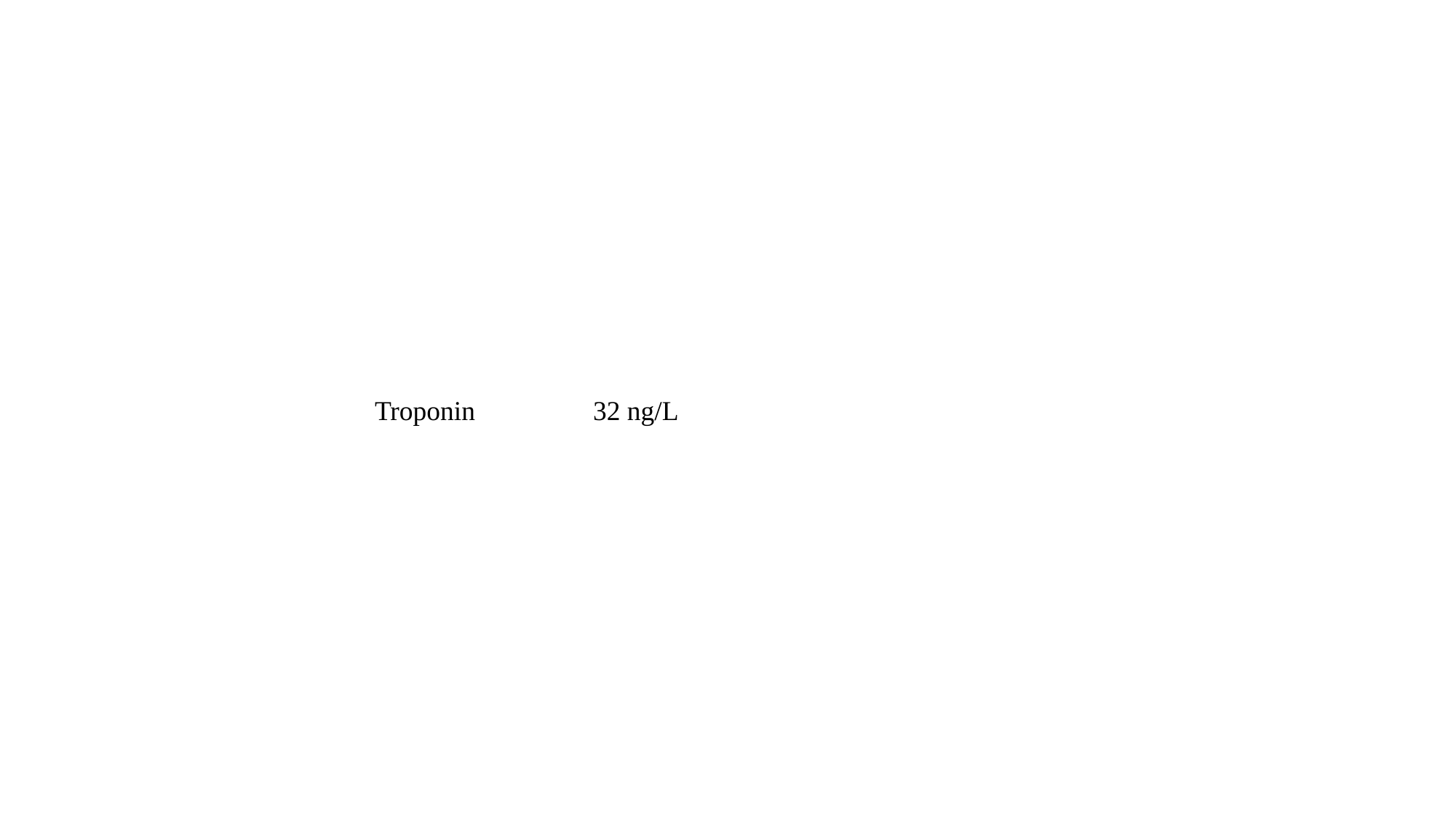

Troponin		32 ng/L

## Slide 7
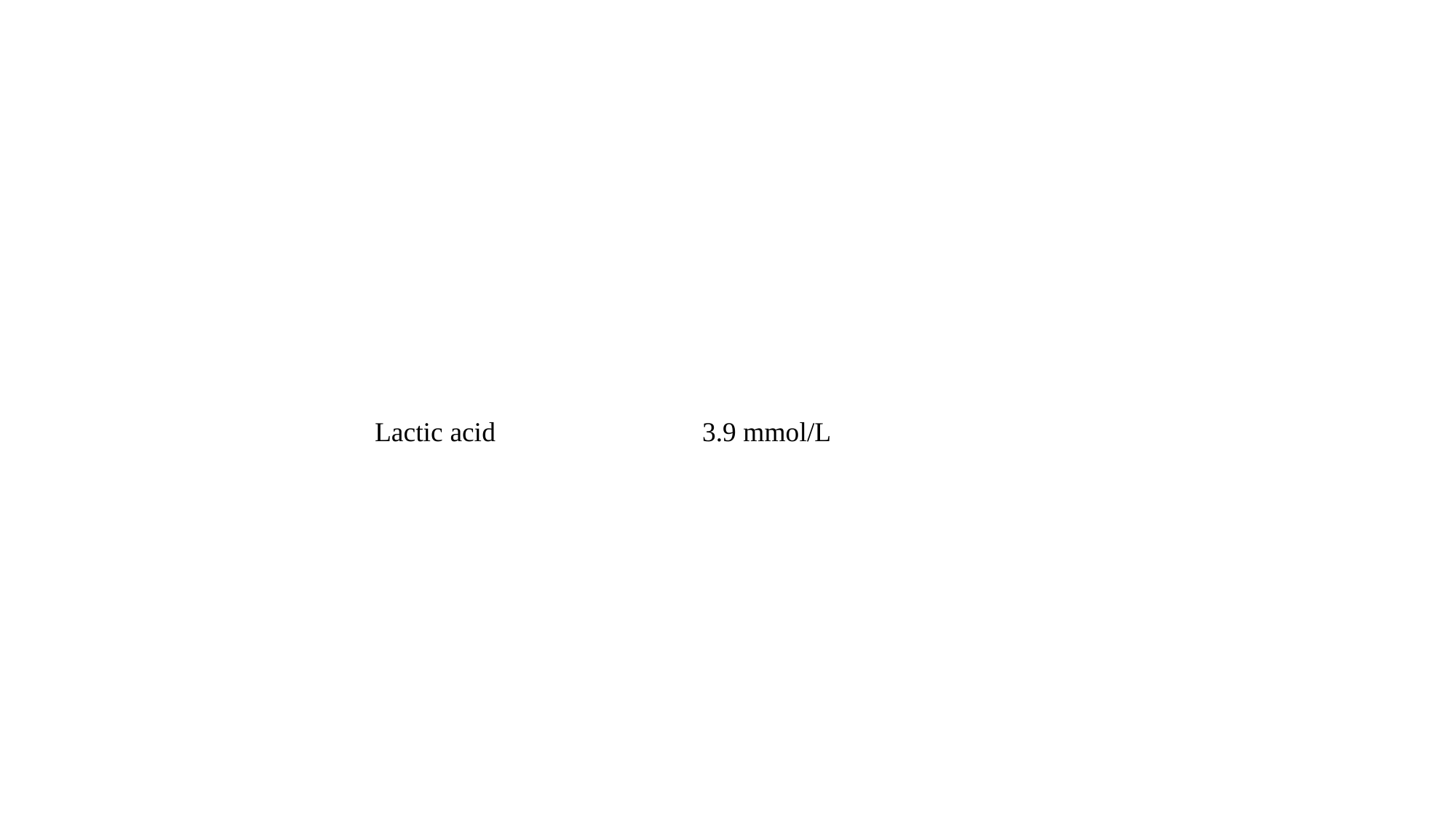

Lactic acid		3.9 mmol/L

## Slide 8
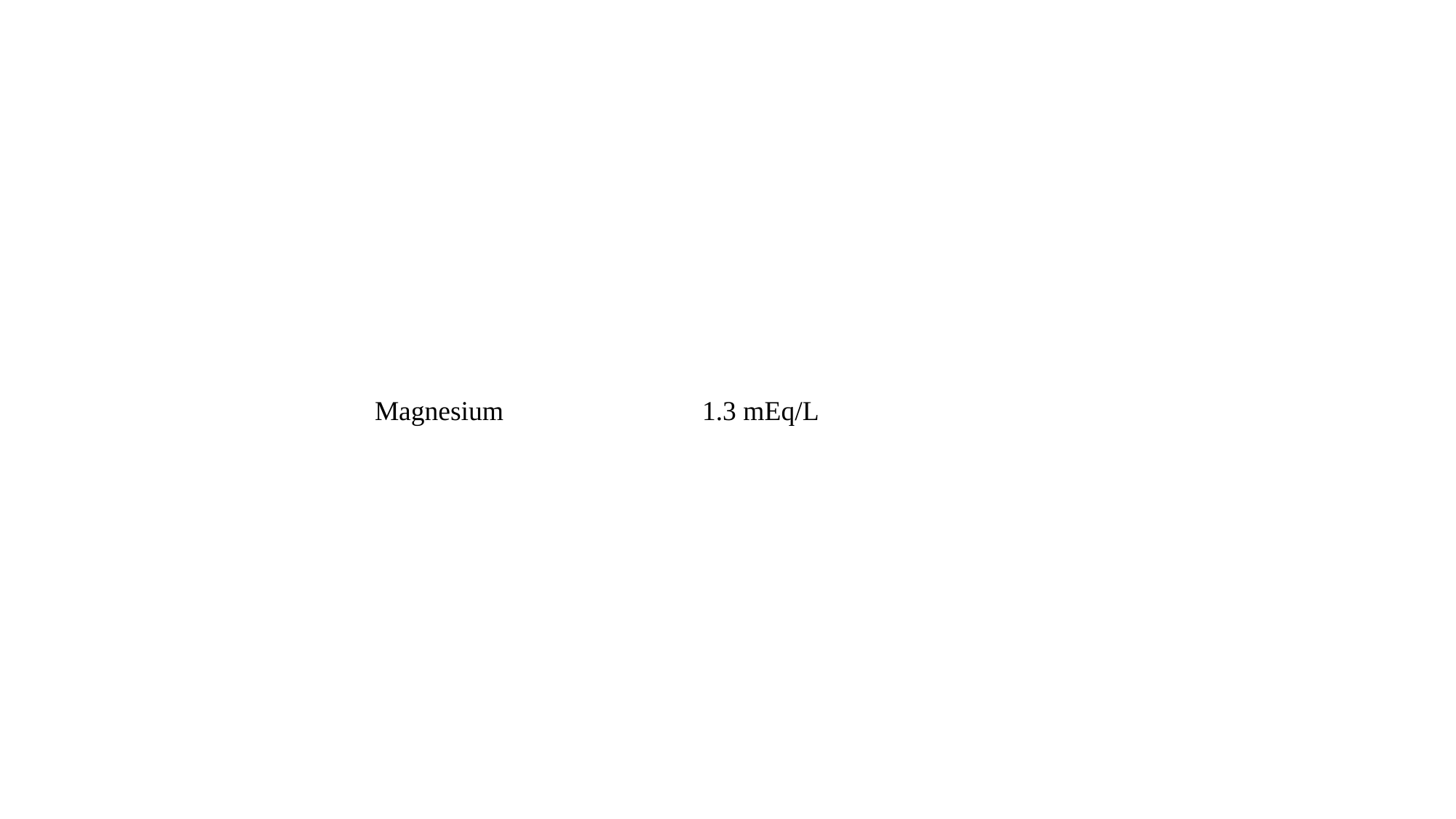

Magnesium		1.3 mEq/L
